# Lifshitz Transition in Correlated Topological Semimetals

**DOI:** 10.1002/advs.202521312

**Published:** 2026-03-24

**Authors:** Byungkyun Kang, Myoung‐Hwan Kim, Chul Hong Park, Anderson Janotti, Eunja Kim

**Affiliations:** ^1^ Department of Physics The University of Texas at El Paso El Paso Texas USA; ^2^ Department of Physics and Astronomy Texas Tech University Lubbock Texas USA; ^3^ Quantum Matter Core‐Facility and Research Center of Dielectric and Advanced Matter Physics Pusan National University Busan Republic of Korea; ^4^ Department of Materials Science and Engineering University of Delaware Newark Delaware USA

**Keywords:** dynamical mean field theory, electron correlations, lifshitz transition, topological semimetals

## Abstract

Topological quasiparticles that arise when the chemical potential is near a band crossing are pivotal to next‐generation quantum devices. Nonetheless, correlated topological materials frequently host Dirac nodes away from the Fermi level, and the cause of this shift remains elusive. Here, we investigated the electronic structure of YPtBi and GdPtBi through ab initio many‐body perturbation GW theory combined with dynamical mean‐field theory and theoretically show that the correlation effects of 4d or 4f electrons can lead to the formation of hole carriers, thereby shifting the quadratic band‐touching points away from the chemical potential. In YPtBi, the weakly correlated Y‐4d electrons constitute the topological bands, and the quadratic band‐touching point is at the Fermi level at high temperatures. At low temperatures, enhanced correlations of Y‐4d electrons renormalize the topological bands, leading to the formation of a hole pocket. In GdPtBi, the strongly correlated Gd‐4f electrons form Hubbard‐like bands that originate from self‐energy effects potentially associated with a topological singularity. These local bands encompass itinerant 4f bands, which hybridize with the topological bands to induce pronounced hole bands. This concerted effect reduces the hole doping, bringing the chemical potential closer to the quadratic band‐touching points as the temperature is lowered. This prediction of temperature‐induced Lifshitz transition can be responsible for the large hole bands observed in both topological semimetals in angle‐resolved photoemission spectroscopy measurements at low temperatures. Our findings indicate that the integration of correlated fermions within a topological framework can reshape the Fermi surface without defect engineering. These theoretical predictions underscore the importance of direct experimental investigation of the temperature‐dependent electronic structure in the topological semimetals.

## Graphical abstract

Theoretical calculations reveal that in YPtBi and GdPtBi, strong electronic correlations shift topological Dirac nodes away from the Fermi level by forming large hole pockets. This effect is sensitive to temperature and arises from interactions among 4d or 4f electrons, offering insights into Fermi surface engineering in quantum materials. These findings highlight the need for experimental studies of temperature‐dependent band structures.

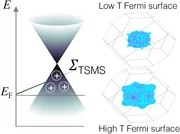

## 1 Introduction

Topological semimetals and topological quasiparticles near Weyl points or quadratic band‐touching points have garnered significant attention due to their intriguing and unconventional physical properties, making them promising candidates for developing robust quantum devices [1, 2, 3, 4, 5, 6, 7, 8, 9, 10]. Research on Weyl fermions has primarily concentrated on weakly correlated electron systems. However, the electronic correlations present in topological systems are renowned for prompting novel electronic properties that are not typically observed in standard metals or insulators. In this context, rare‐earth‐based (including yttrium) topological half‐Heusler semimetals are suggested to display exotic physics, including tunable normal‐state band inversion strength, superconducting pairing, magnetically ordered ground states, unusual topological surface states, anomalous Hall effects, heavy fermion behavior, quantum criticality, and Weyl fermions in strongly correlated electron systems [11, 12, 13, 14, 15, 16, 17, 18, 19, 20, 21, 22, 23, 24, 25, 26, 27, 28, 29].

In RPtBi (R = rare earth element and Y), the topology of the Fermi surface is highly sensitive to the choice of R, significantly influencing exotic quantum phenomena [25, 30, 31]. One proposed scenario for the impact of R‐4f electrons on topological bands at the Fermi level involves the Kondo effect. It is suggested that electronic bands near the Fermi level may become heavily renormalized due to the strong Kondo coupling between R‐4f electrons and conduction band states below the Kondo temperature, which significantly affects the topologically non‐trivial state in ${\mathrm{SmB}}_{6}$ [32, 33, 34]. For the Kondo effect to occur, f electrons must be close to the Fermi level. However, the varying energy levels of quadratic band‐touching points in RPtBi are not explained by the Kondo effect alone. Specifically, angle‐resolved photoemission spectroscopy (ARPES) measurements of GdPtBi at 15 K, which features a strongly localized 4f electron shell far from the Fermi level and large hole bands, have deduced quadratic band‐touching points at 0.4 eV above the Fermi level, with no indication of a saddle point near the Fermi level [35]. In contrast, longitudinal magnetoresistance experiments [15] indicate the existence of the chiral anomaly in p‐type GdPtBi samples at 2.5 K. This observation relies on a model featuring quadratic band‐touching points near the Fermi level. When a magnetic field of B = 6 T is applied, the Zeeman splitting positions the chemical potential between saddle points, which is essential for the occurrence of the chiral anomaly. The band structure calculations of GdPtBi, using density functional theory combined with Hubbard U (DFT+U), showed that the quadratic band‐touching points are located precisely at the Fermi level [15, 16]. In the research, the splitting of these quadratic bands remains below 0.1 eV, when a magnetic field of B = 8 T is applied [16]. This indicates that the quadratic band‐touching points identified at 0.4 eV by the ARPES [35] are unlikely to develop into a chemical potential between saddle points through the Zeeman splitting. Crystallographic defects in the topological semimetal have the potential to induce charge doping and a hole band [6, 36]. However, evidence for the formation of a large hole band, as measured by ARPES, remains elusive in GdPtBi because it would require a high concentration of defects. Besides of the R‐4f, ARPES measurements of YPtBi reveal that the quadratic band‐touching point lies above the Fermi level and a large hole band is present [26, 27], indicating a significant impact of Y‐4d electrons on the topological bands structure. These findings suggest that the electronic structure of RPtBi near the Fermi level is unclear, indicating that an unknown many‐body effect associated with temperature impacts the system.

**FIGURE 1:**
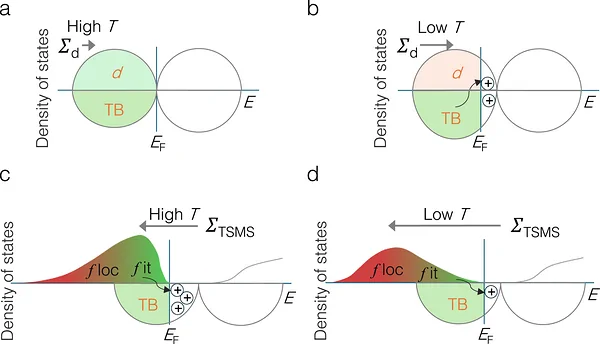
Schematic diagram of the impact of electron correlations on the formation of topological hole bands. (a) In YPtBi, the Y‐4d orbitals contribute the topological bands (TB), along with other orbitals, there is no significant self‐energy of Y‐4d at high temperature. In this case, the quadratic band‐touching points (QTP) are at the chemical potential ($E_{F}$). (b) In YPtBi, the enhanced self‐energy of Y‐4d at low temperature renormalizes the TB, including Y‐4d bands. This increase density of states of the occupied TB leading to the formation of hole bands. (c) In GdPtBi, the topological singularity‐induced Mott‐like self‐energy (TSMS, horizontal arrow) at the QTP gives rise to broadened Hubbard‐like bands, which comprise the 4f local bands (f loc) and the 4f itinerant bands (f it). These bands act as hole dopants for the TB, shifting the $E_{F}$ downward from the QTP. At high temperatures (High T), TSMS pushes the Hubbard‐like bands away from the QTP, with an overlap between f it and TB, which leads to hole doping and $E_{F}$ shifting. (d) In comparison to High T, at Low temperature (Low T), the TSMS strengthens, pushing the Hubbard‐like bands further from the QTP. This reduces the overlap between f it and TB, diminishes hole doping of the TB, and leads to a smaller shift of $E_{F}$ away from the QTP.

Recent studies in quantum many‐body systems have shown that the dynamic properties of R‐4f electrons vary considerably depending on the specific class of R‐based materials. In the case of the superconductor ${\mathrm{NdNiO}}_{2}$, Nd‐4f electrons are strongly localized, resulting in the Kondo effect [37]. In contrast, Ce‐4f electrons in Ce films exhibit itinerancy, forming dispersive quasiparticle bands [38]. Research by Park et al. [39] illustrates that the single 4f electron in cerium within ${\mathrm{CeRhIn}}_{5}$ simultaneously contributes to magnetism, a characteristic of localization, and superconductivity, which requires itinerancy. Our recent study [40] proposed that the screening suppression of R‐4f at topological singular points in RPtBi and RAlGe prompts a robust topological singularity‐induced Mott‐like self‐energy (TSMS). In this study, we theoretically predict that GdPtBi exhibits an interplay of the dual nature of Gd‐4f and the proposed TSMS. As illustrated in Figure 1c,d, the TSMS can gives rise to Gd‐4f Hubbard‐like local bands, which incorporate the Gd‐4f itinerant bands. The latter hybridizes with topological bands, leading to topological hole bands. The Hubbard‐like bands are pushed down from the quadratic band‐touching points by TSMS, which becomes more pronounced at lower temperatures. This interplay leads to a Lifshitz transition in GdPtBi. In contrast, as shown in Figure 1a,b, the Y‐4d orbitals contribute to the formation of topological bands in YPtBi, along with other orbitals. The enhanced self‐energy of the Y‐4d at low temperatures leads to the emergence of topological hole bands.

To account for the interplay between electron correlation and topological bands, we ascertain the electronic structure of GdPtBi, YPtBi, and YbPtBi using the ab‐initio linearized quasiparticle self‐consistent GW combined with the dynamical mean field theory (LQSGW+DMFT) approach [41]. This method allows us to precisely determine the temperature‐dependent electronic structure of correlated materials without any subjective biases. While we adopt the experimental lattice parameters [42], we explicitly calculate all other parameters, including the double‐counting energy and the Coulomb interaction tensor. Further details can be found in the Methods section. The LQSGW+DMFT approach has been applied to study many‐body effects, including Kondo effects [37, 43, 44, 45, 46], Hund and Mott physics [47, 48], and interplay between strong electronic correlations and nontrivial topology [49].

## 2 Temperature‐Induced Lifshitz Transition in YPtBi

**FIGURE 2:**
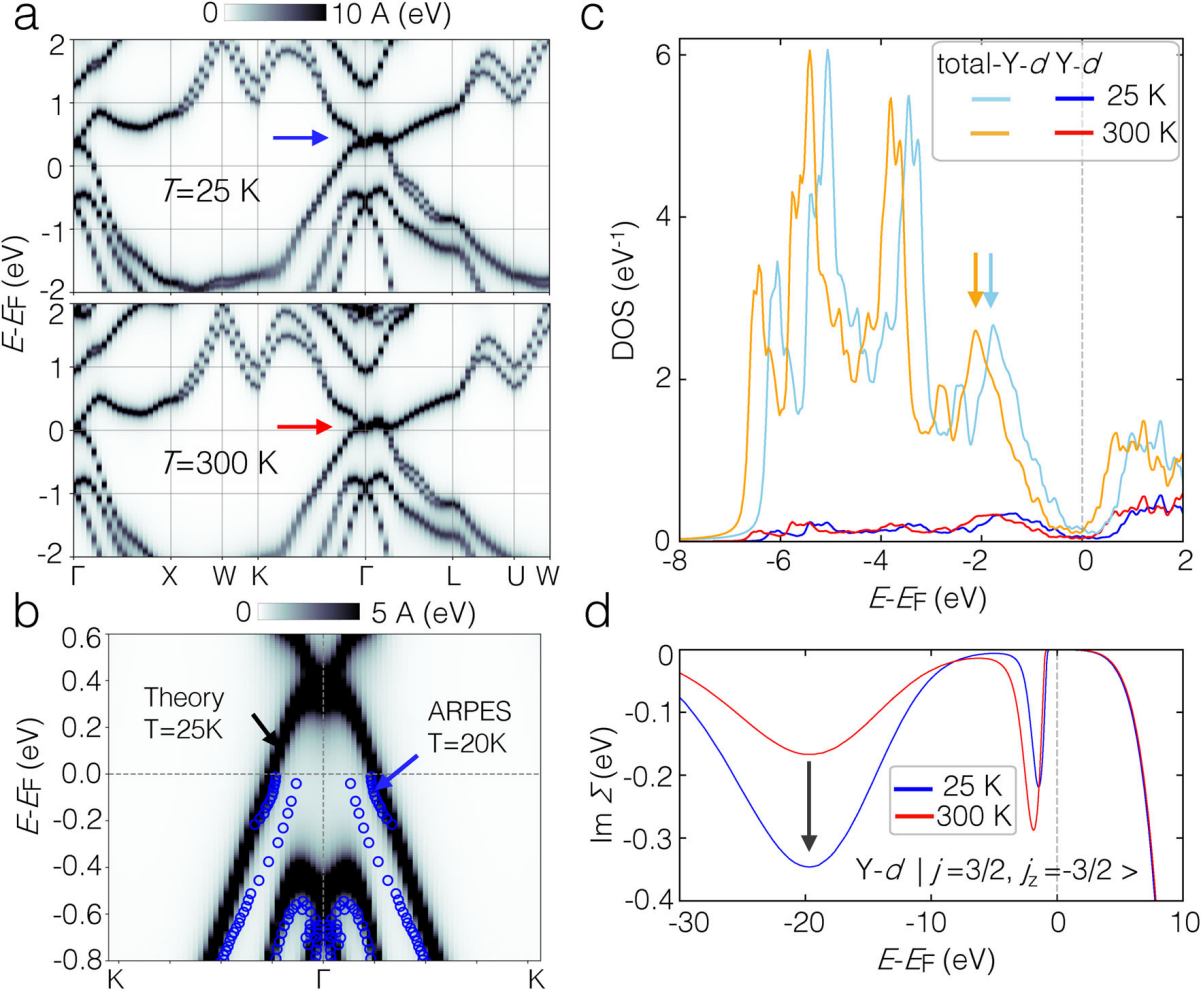
Electronic structure of YPtBi. (a) Calculated spectral functions at T=25 K (top panel) and T=300 K (bottom panel). The energy position of the quadratic band‐touching points aligns with the Fermi level at 300K (highlighted by the red arrow), whereas at 25K, the quadratic band‐touching points are located approximately 0.4 eV above the Fermi level (indicated by the blue arrow). (b) Calculated spectral function at T=25 K along the K‐Γ‐K high‐symmetry line. The electronic structure of the (111) surface of YPtBi, measured by ARPES at T = 20 K [26], was extracted and is indicated by blue dots. (c) Density of states projected onto the Y‐4d orbitals, as well as the total density of states with the Y‐4d contribution subtracted, calculated at both 25 and 300K. The temperature‐dependent shift in the DOS peak is indicated by sky blue arrows (25 K) and orange arrows (300 K). (d) Imaginary part of the self‐energy for the Y‐4d state with $|j=3/2,j_{z}=-3/2\rangle$. The enhancement of the self‐energy at 25 K relative to 300 K is highlighted by the black arrow.

We first report the emergence of hole bands in YPtBi, where the R‐4f state is unoccupied, in contrast to GdPtBi and YbPtBi. Figure 2a shows the calculated spectral functions of YPtBi. The presence of quadratic band‐touching points near the Fermi level at the Γ point is apparent at both T=300 K and T=25 K. At 300 K, these quadratic band‐touching points are located at the Fermi level, whereas at 25 K, they are shifted to approximately 0.4 eV above the Fermi level. In Figure 2b, the spectral function along the K‐Γ‐K high‐symmetry direction at 25 K is compared with ARPES measurements performed at 20 K [26]. The ARPES data display a strong, disconnected linear feature with a weaker linear branch near the Fermi level, while our calculations reveal a prominent single linear dispersion intersecting the Fermi level. Notably, both ARPES measurements and theoretical calculations demonstrate the absence of quadratic band‐touching points below the Fermi level.

The Y‐4d electrons in YPtBi exhibit weak electronic correlations, as evidenced by the comparatively small value of the static on‐site Coulomb interaction (obtained using cRPA; see Figure S1), $U_{C}\left(\omega =0\right)=1.9$ eV, and the large electronic bandwidth of approximately 7 eV (Figure 2c). Consequently, Y‐4d electrons contribute to the topological bands together with other electronic states in YPtBi, as illustrated in Figure 2c. These findings suggest that the Y‐4d electrons in YPtBi may be described as forming an electron gas, with their correlation effects potentially exhibiting temperature dependence [50].

In YPtBi, the self‐energy associated with the Y‐4d states, which is centered below the topological bands at approximately –20 eV, exhibits a marked increase as the temperature is reduced from 300 K to 25 K, indicative of enhanced electron correlations within the Y‐4d orbitals at low temperatures, as shown in Figure 2d. The enhancement in self‐energy at 25 K raises the energy of the Y‐4d electrons. Furthermore, strong hybridization between the Y‐4d electrons and the topological bands shifts the latter upward, which in turn lowers the chemical potential and renormalizes the topological bands, increasing the density of states below the Fermi level.

The LQSGW+DMFT methodology achieves self‐consistency through an iterative loop involving the ComLowH and ComCTQMC modules [41]. In each cycle, ComLowH computes the lattice Green's function by incorporating both the non‐local LQSGW Hamiltonian ($H_{QP}^{\mathrm{nl}}$) and the local impurity self‐energy (${\tilde{\Sigma }}_{imp}$), thereby generating the Weiss field ($\tilde{G}$). Using this Weiss field, ComCTQMC determines the chemical potential and updates the impurity self‐energy. This unified treatment of local and non‐local interactions within the lattice Green's function allows for self‐consistent adjustment of the chemical potential and self‐energy, facilitating physically realistic charge transfer between eigenstates while enforcing charge neutrality. The enhancement of the self‐energy for Y‐4d states facilitates charge transfer from the Y‐4d orbitals to the topological bands (total states minus Y‐4d contribution). Although this charge transfer is relatively small, it is evident in the calculated Y‐4d occupancy, which decreases from 1.15 at 300 K to 1.09 at 25 K. These occupation numbers are obtained by applying the occupation operator in Matsubara frequency within the ComCTQMC module. Currently, determination of the occupation for uncorrelated orbitals, such as Bi‐p, within the full space of the lattice Green's function is not implemented in LQSGW+DMFT. As a result, the analysis of charge transfer is inferred from the DOS calculated on the real frequency axis, a procedure that may introduce some error due to the analytic continuation process. The peak in the (total ‐ Y‐4d) DOS—primarily comprising Pt‐5d and Bi‐6p orbitals, as shown in Figure S5—shifts to a higher intensity near –1.8 eV at 25 K, compared to the corresponding peak near –2.2 eV at 300 K, as illustrated in Figure 2c. This behavior, therefore, qualitatively suggests charge transfer from the Y‐4d orbitals to the topological bands. These findings indicate that the increase in DOS within the topological bands (total ‐ Y‐4d) at lower temperatures predominantly contributes to effective hole doping.

## 3 Topological Singularity‐Induced Mott‐Like Self‐Energy of R‐4*f*

Whereas the weakly correlated Y‐4d states in YPtBi lead to modest renormalization of the topological bands, the R‐4f electrons exhibit strong correlations with the topological bands. Our recent work [40] theoretically predicts that TSMS may emerge in HoPtBi, PrPtBi, and PrAlGe using the LQSGW+DMFT approach. Generally, for Ho‐4f electrons, a moderate onsite Coulomb interaction (U=2.03 eV) supports quasiparticle formation at low temperatures. However, at topological singularities, the nearly vanishing DOS associated with the quadratic band‐touching point of topological bands at the Fermi level leads to a pronounced suppression of the 4f hybridization function and thus of screening. This results in a significantly enhanced effective U, approaching the bare Coulomb interaction value, which in turn drives the formation of divergent Mott‐like self‐energy at the quadratic band‐touching point. Consequently, instead of well‐defined quasiparticles at the Fermi level, the 4f states manifest Hubbard‐like broadened incoherent spectral features and half‐filling, that are hallmarks of Mott physics.

To model the divergent TSMS, we introduce schematic perturbative self‐energy diagrams for the 4f electrons, comprising all irreducible local contributions [40]. At the topological singularity, the suppression of screening eliminates higher‐order particle‐hole bubble diagrams, resulting in a self‐energy dominated by the bare Coulomb interaction, analogous to conventional Mott physics. A comparative analysis of the self‐energy magnitude among HoPtBi and GdPtBi (TSMS), NiO (Mott) [51], ${\mathrm{LaNiO}}_{2}$ (Hund) [52], and ${\mathrm{UTe}}_{2}$ (Kondo) [45] shows that both TSMS and Mott systems exhibit divergent self‐energy behavior. In contrast, the Hund and Kondo systems exhibit considerably weaker electronic correlations, as indicated by the much less pronounced self‐energy enhancements [40].

Previous work has shown that quadratic band‐touching in GdPtBi can give rise to Weyl nodes under an applied magnetic field, as predicted by density functional theory (DFT) [15]. Our LQSGW+DMFT calculations yield band structures near the quadratic band‐touching point that are in good agreement with DFT predictions. Linear band crossings, such as type‐I Weyl nodes observed in PrAlGe, can also induce TSMS due to the low DOS at the nodal point [40]. Collectively, these findings indicate that TSMS are not determined by the detailed topological properties themselves, but are generally characterized by the presence of a near‐zero DOS at the singular point.

## 4 Temperature‐Induced Lifshitz Transition in GdPtBi

**FIGURE 3:**
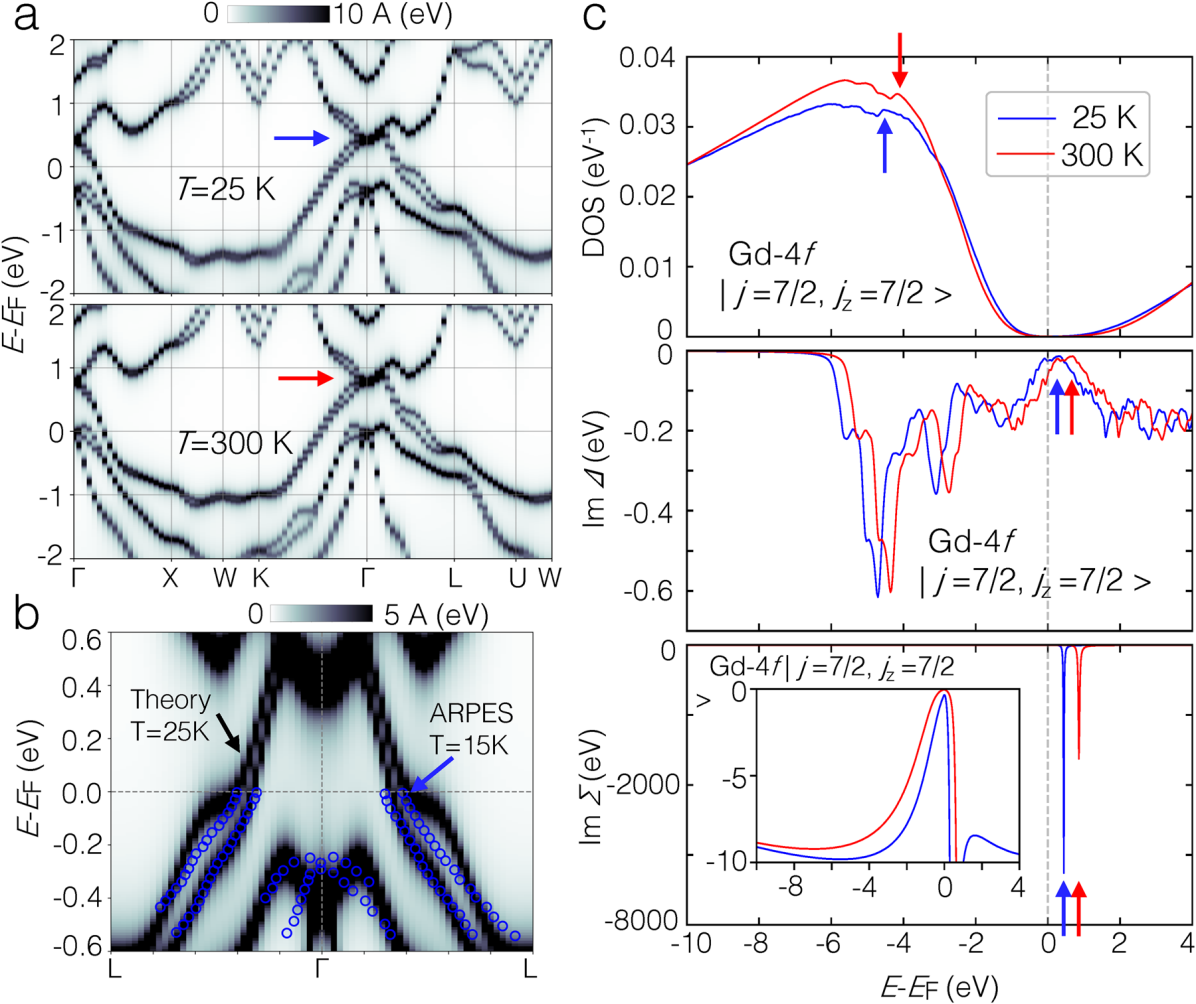
Electronic structure of GdPtBi. (a) Calculated spectral functions at T=25 K (upper panel) and T=300 K (lower panel). The energy levels of the quadratic band‐touching points are located at approximately 0.8 eV (indicated by the red arrow) at 300 K and approximately 0.4 eV (indicated by the blue arrow) at 25 K. (b) Calculated spectral function at T=25 K (indicated by the black arrow) along the L‐Γ‐L high‐symmetry direction. The experimentally measured band structure at T=15 K, obtained by ARPES [35], is extracted and shown as blue dots. By performing a linear extrapolation of the ARPES‐measured bands, a Dirac point is predicted at approximately 0.4 eV above the Fermi level, which is consistent with our theoretical calculations. (c) Projected DOS, imaginary part of the hybridization function (Δ), and the imaginary part of the self‐energy of the Gd‐4f state corresponding to $|j=7/2,j_{z}=7/2\rangle$. Temperature‐dependent shifts of the DOS peak, the minimum of the hybridization function, and the self‐energy are indicated by blue arrows (25 K) and red arrows (300 K).

We calculated temperature‐dependent hole bands in GdPtBi, which can be explained by invoking the presence of TSMS. However, definitive proof of TSMS requires further investigation, as discussed in the Discussion section. Figure 3a presents the calculated spectral functions of GdPtBi. The quadratic band‐touching points near the Fermi level at the Γ point are evident at both T = 300 K and T = 25 K. At 300 K, the quadratic band‐touching points are observed at ∼0.8 eV, shifting to ∼0.4 eV at 25 K. The evolution of the Fermi surface with temperature (see Figure S3) is reminiscent of the temperature‐induced Lifshitz transition in the topological insulator ${\mathrm{ZrTe}}_{5}$ [53].

The spectral function at 25 K along the L‐Γ‐L high‐symmetry line is compared with ARPES measurements at 15 K [35] in Figure 3b. Although ARPES measurements reveal linear band dispersions near the Fermi level in GdPtBi, the computed spectral function does not exhibit a clear linear dispersion in the presented energy range. In contrast, for YPtBi (Figure 2), where the 4f electrons are absent in energy relative to the Gd‐4f states in GdPtBi, both experimental and theoretical results show nearly linear bands below the Fermi level. This suggests that the presence of Gd‐4f electrons in GdPtBi alters the electronic structure near the Fermi level, leading to the deviations from linearity observed in the calculated spectral function. Notably, the approximate band crossing point of 0.4 eV, deduced from the ARPES measurements [35], aligns well with the calculated quadratic band‐touching points in the spectral function.

The temperature‐induced Lifshitz transition in GdPtBi is attributed to the interplay of the dual nature of Gd‐4f electrons with TSMS. The calculated static $U_{C}\left(\omega =0\right)$ of Gd‐4f in GdPtBi is 3.4 eV. In comparison to the Mott‐like self‐energy of the Pr‐4f in PrPtBi, which is ∼–8 eV and is attributed to a larger on‐site Coulomb interaction $U_{C}\left(\omega =0\right)=6.3$ eV for Pr‐4f, as reported in Ref. [40], GdPtBi exhibits a substantially greater self‐energy for the Gd‐4f, on the order of ∼–80 eV, despite having a smaller $U_{C}\left(\omega =0\right)$. This indicates a distinct correlation mechanism underlying the electronic structure of the Gd‐4f states in GdPtBi compared to the Pr‐4f states in PrPtBi. Therefore, the divergent self‐energy peak observed, as depicted in Figure 3c, can be interpreted as TSMS. This arises from the concerted effect between R‐4f quasiparticles and the topological singularity, as demonstrated in our work [40]. Gd‐4f, being subject to intermediate Coulomb repulsion, has a higher probability of forming quasiparticles near the Fermi level at lower temperatures [37, 54]. This leads to increased screening suppression at the quadratic band‐touching points near the Fermi level, resulting in enhanced TSMS at 25 K, as illustrated in the self‐energy plot in Figure 3c. Figure 3c illustrates the DOS, hybridization function, and self‐energy of the Gd‐4f state of the $|j=7/2,j_{z}=7/2\rangle$. The strong TSMS induces lower Hubbard‐like incoherent bands in the DOS, a hallmark of Mott physics that indicates the localized behavior of correlated electrons. Small peaks (or shoulders) in the DOS appear at approximately –4.0 and –2.3 eV at 300 K, shifting to –4.4 and –2.7 eV at 25 K, respectively. There is no sizable peaks in the self‐energy around these energy level as shown in inset in Figure 3c. These peaks, therefore, can be associated with the major peaks in the Gd‐4f hybridization functions at the corresponding energy levels, suggesting an interaction between the Gd‐4f state and the topological bands. These findings imply that the Gd‐4f state in GdPtBi possesses a dual character, exhibiting both localized and itinerant electron behavior.

As shown in Figure 3c, the hybridization function maintains a consistent magnitude across both temperatures, demonstrating that Gd‐4f electrons remain fully hybridized with the topological bands irrespective of temperature. Consequently, Gd‐4f electrons effectively introduce hole doping to the topological bands. This interaction pushes the chemical potential down from the quadratic band‐touching points, consistently observed at both temperatures. The extent of self‐doping is influenced by the strength of TSMS. At 25 K, compared to 300 K, an increase in TSMS causes the Gd‐4f Hubbard‐like bands to move further from the quadratic band‐touching points. This shift reduces the Gd‐4f DOS between –6 eV and the quadratic band‐touching points, where hybridization occurs, and subsequently decreases the hole doping effect on the topological bands, adjusting the chemical potential closer to the quadratic band‐touching points. This behavior contrasts with that observed in LaPtBi, where the quadratic band‐touching points align with the Fermi level in the absence of 4f electrons [40]. Moreover, this is different compared to the temperature‐dependent Nd‐4f bands, as they do not affect the chemical potential of ${\mathrm{NdNiO}}_{2}$ [37]. The degree of self‐hole doping, or the shift in the chemical potential of the topological bands, is dependent on the extent to which the Gd‐4f Hubbard‐like bands overlap with the topological bands.

## 5 Lifshitz Transition is Absent for Yb‐4f and Present for Yb‐4d in YbPtBi

**FIGURE 4:**
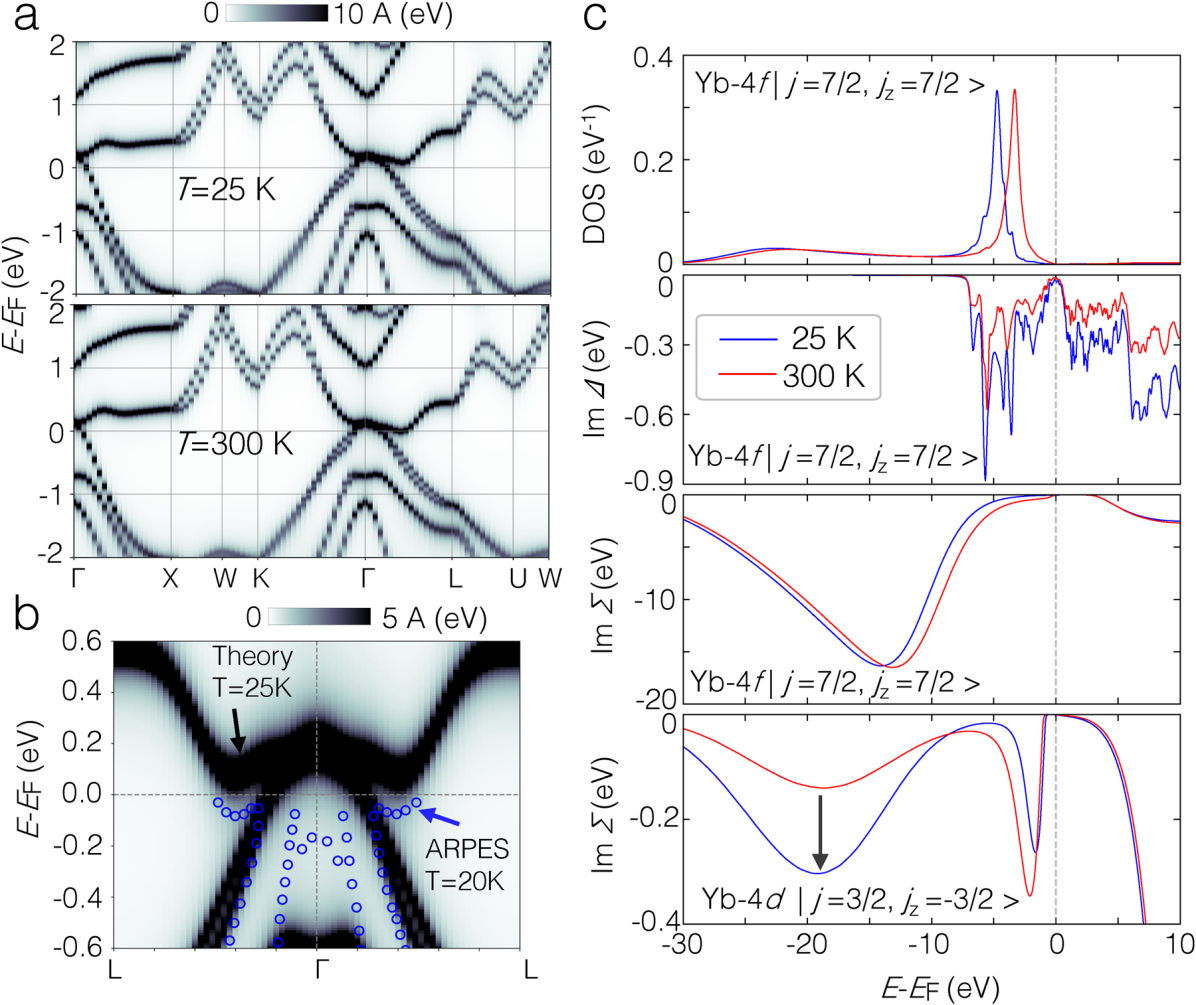
Electronic structure of YbPtBi. (a) Spectral functions at T = 25 K (upper panel) and T = 300 K (lower panel). (b) Calculated spectral function at T = 25 K, along the L‐Γ‐L high symmetry line. ARPES measurements at T = 20 K [14] are denoted by blue dots. (c) Projected DOS, the imaginary part of the hybridization function (Δ) and imaginary part of the self‐energy of the Yb‐4f state of the $|j=7/2,j_{z}=7/2\rangle$. The enhancement of the Yb‐4d self‐energy at 25 K relative to 300 K is highlighted by the black arrow.

Unlike YPtBi and GdPtBi, YbPtBi exhibits quadratic band‐touching points near the chemical potential, which we investigated in terms of correlations effects of the Yb‐4f and Yb‐4d states. Figure 4a presents the calculated spectral functions of YbPtBi. It shows quadratic band‐touching points at the Γ point, positioned in the vicinity of the Fermi level, in contrast to those in GdPtBi. Unlike GdPtBi and YPtBi, YbPtBi exhibit a small chemical potential shift when lowering the temperature from 300 to 25 K. The spectral function at 25 K, along the L‐Γ‐L high symmetry line, is compared with ARPES measurements taken at 20 K [14] in Figure 4b. Both the theoretical and experimental results manifest two hole pockets crossing the Fermi level and doubly degenerate $\lambda_{6}$ electron pockets near the Fermi level. Our results agree with DFT simulations in which Yb‐4f electrons are treated as core states [14, 55]. This indicates that the strong many‐body effect observed in GdPtBi is absent in YbPtBi, and the 4f electrons in YbPtBi do not affect the topological bands near the Fermi level.

Figure 4c shows the DOS, hybridization function, and self‐energy of the Yb‐4f state of the $|j=7/2,j_{z}=7/2\rangle$. The fully filled Yb‐4f states are positioned far below the Fermi level in the DOS. However, small peaks at ∼–4 and ∼–6 eV in both the DOS and hybridization functions at 25 K indicates that the Yb‐4f states also hybridized with topological bands. In contrast to GdPtBi, there is no divergent self‐energy attributed to TSMS in YbPtBi. Although cooling causes the Yb‐4f state to further distance themselves from the Fermi level, YbPtBi does not demonstrate significant temperature‐induced changes in topological bands from ‐2 to 2 eV, as shown in Figure 4a. This is because the interaction between the 4f and topological bands is too localized to induce the Lifshitz transition. However, the small chemical potential shift is attributed to the enhanced self‐energy of Yb‐4d see the bottom panel of Figure 4c, similarly to YPtBi.

## 6 Impact of Topological Singularity‐Induced Mott‐Like Self‐Energy

**FIGURE 5:**
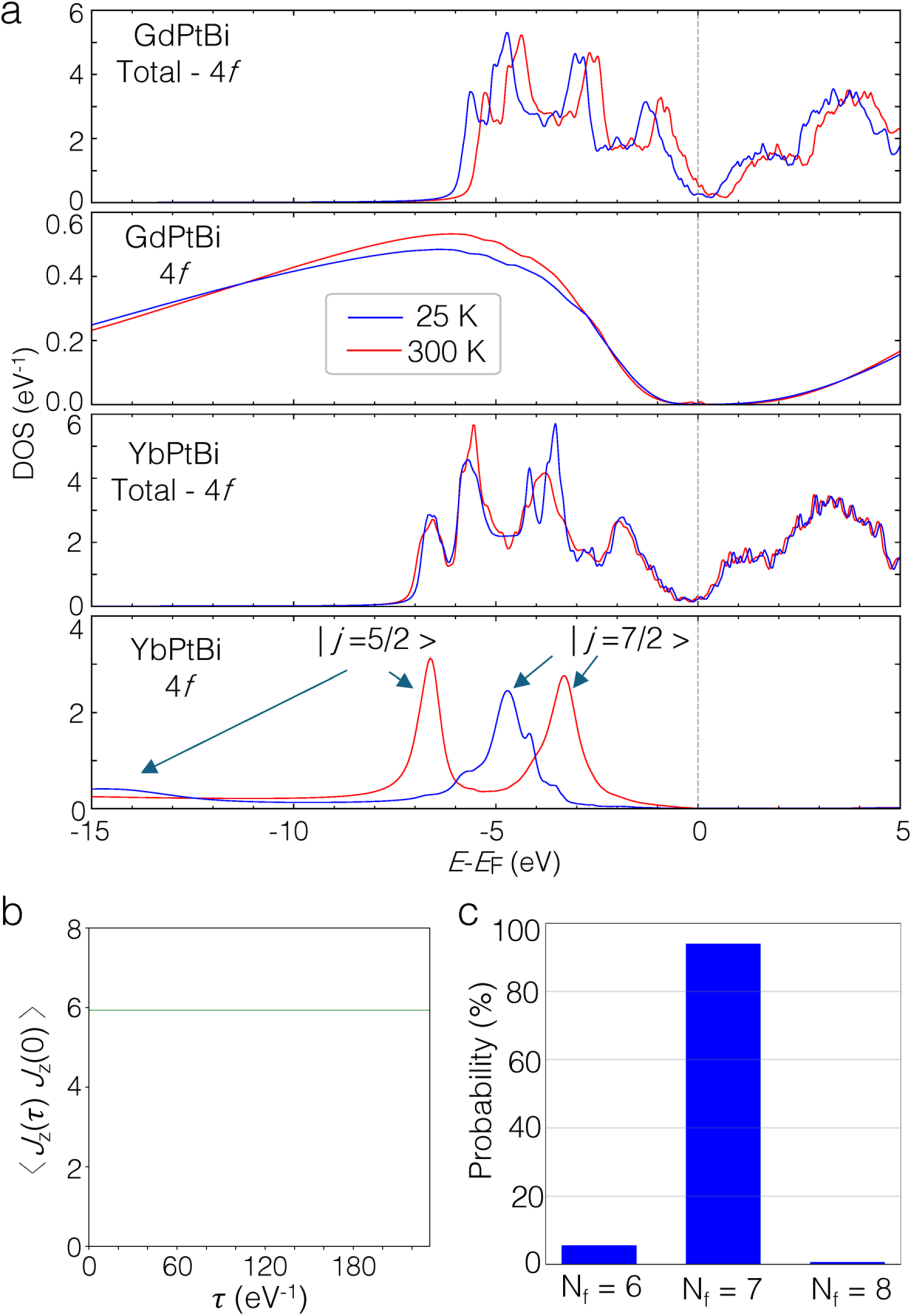
Comparison of electronic structures. (a) The calculated DOS of GdPtBi: total DOS minus the 4f contribution (first panel) and the 4f contribution only (second panel) at 25 and 300 K. Similarly, the calculated DOS of YbPtBi: total DOS minus the 4f contribution (third panel) and the 4f contribution only (fourth panel) at 25 and 300 K. (b) The local spin moment correlation function in the imaginary time τ of the Gd‐4f in GdPtBi at 25 K. (c) Valence histograms of the Gd‐4f in GdPtBi at 25 K.

To further elucidate the impact of the predicted TSMS on the temperature dependent Lifshitz transition in RPtBi, a comparative analysis was conducted on the DOS of GdPtBi with YbPtBi, the latter of which lacks the TSMS. As shown in Figure 5, the Yb‐4f DOS in YbPtBi exhibits local peaks centered at ∼−3.3 eV for the j=7/2 multiplet and at ∼−6.7 eV for the j=5/2 multiplet at 300 K. The j=7/2 multiplet states are predominantly hybridized with topological bands (total ‐ 4f DOS) at ∼−4.0 eV. This is evident from the smaller DOS of the topological bands compared to that at 25 K, where the j=7/2 multiplet states have shifted toward lower energy. Due to the shift at 25 K, the j=7/2 multiplet states hybridize at ∼−6.0 eV, resulting in a smaller DOS for the topological bands at ∼−6.0 eV compared to that at 300 K. Although these temperature‐dependent hybridizations alter the topological bands locally, they do not change the energy levels of the topological bands. This is attributed to the strongly localized DOS of Yb‐4f electrons, which can be treated as core electrons [55].

However, the character of Gd‐4f in GdPtBi differs from the localized Yb‐4f in YbPtBi. The local Green's function in DMFT [56] is expressed as $G_{loc}^{-1}\left(i\omega_{n}\right)=i\omega_{n}+\mu -\Delta \left(i\omega_{n}\right)-\Sigma \left(i\omega_{n}\right)$ on the imaginary frequency, where μ is the chemical potential, $\Delta (i\omega_{n})$ represents the hybridization function, and $\Sigma (i\omega_{n})$ is the impurity self‐energy. In GdPtBi, self‐energy is governed by TSMS. At low temperatures, Gd‐4f quasiparticles are highly formed at μ if there is no TSMS. When μ is at the quadratic band‐touching points, due to the incompatibility between the 4f quasiparticle and the topological singular point at the same energy level [40], a diverging TSMS appears at μ, leading the quasiparticles to significantly contribute to the Hubbard‐like Gd‐4f spectral function. Then, the $\Delta (i\omega_{n})$, arising from the dual nature, affects the $G_{\text{loc}}\left(i\omega_{n}\right)$ by shifting μ downward, away from the quadratic band‐touching points. The changing μ, instead of altering $G_{loc}\left(i\omega_{n}\right)$ locally as in YbPtBi, is attributed to the strong TSMS, which leads to the incoherent broadening of the Hubbard‐like Gd‐4f DOS. This hybridizes with most of the topological bands, unlike YbPtBi, as shown in Figure 5. Although the TSMS‐induced Gd‐4f DOS broadens from the chemical potential to beyond ‐15 eV, Gd‐4f is strongly localized, i.e., Hubbard‐like. The local spin moment correlation function, $\chi_{J_{Z}}\left(\tau \right)=\left\langle J_{z}\left(\tau \right)J_{z}\left(0\right)\right\rangle$, is presented in Figure 5b. This correlation function can be used to evaluate the extent of magnetic moment localization [57]. The $\chi_{J_{Z}}\left(\tau \right)$ of Gd‐4f in GdPtBi is found to remain constant with an increase in τ, suggesting the presence of strong local magnetic moments. This is reminiscent of the strong local spin moment found in the van der Waals 2D Mott‐insulator FeSe [58], in the periodic Anderson model during its metal–insulator transition [59], in iron and nickel at ambient and high pressure [60], in heterogeneous Ta‐dichalcogenide bilayers [61], in strongly correlated Hund metals [62], and in Fe‐based superconductors [63]. Additionally, as shown in Figure 5c, the valence histogram of the Gd‐4f in GdPtBi exhibits subtle valence fluctuations, indicating that the local character of Gd‐4f dominates.

## 7 Discussion

We show that the quadratic band‐touching points in GdPtBi are located at ∼0.4 eV above the Fermi level at 25 K, which agrees with the metallic Fermi surface measured by ARPES at 15 K [35]. We also show that the chemical potential shifts from ∼0.8 to ∼0.4 eV, moving closer to the quadratic band‐touching points as the temperature decreases from 300 to 25 K, due to enhanced TSMS at lower temperatures. At present, 25 K represents the lowest temperature accessible in LQSGW+DMFT simulations, owing to the substantial computational cost and the onset of the sign problem in quantum Monte Carlo simulations at lower temperatures. Nevertheless, our calculations suggest that further cooling may draw the chemical potential nearer to the quadratic band‐touching points, aligning the Fermi level between the saddle points as a result of Zeeman splitting of 0.1 eV when a magnetic field is applied [16]. Therefore, the theoretically predicted temperature‐induced Lifshitz transition may play a crucial role in the emergence of the chiral anomaly in p‐type GdPtBi samples, as measured at T = 2.5 K [15].

As a possible cause of the predicted temperature‐dependent Lifshitz transition in GdPtBi, we proposed TSMS, which is supported by the divergent self‐energy behavior. However, it should be emphasize that the self‐energy phenomena discussed here provide indirect evidence and are not directly experimentally measurable, thus requiring further investigation to establish the presence of TSMS conclusively. A detailed minimal model or a comprehensive topological analysis—including the explicit interaction with the f band that could account for such a self‐energy divergence—is beyond the scope of the current work and will be the subject of future studies.

In summary, in the weakly correlated topological semimetal YPtBi, the observed hole bands are attributed to a temperature‐dependent Lifshitz transition resulting from enhanced electron correlation of Y‐4d electrons. In the strongly correlated topological semimetal GdPtBi, the Gd‐4f electrons, subjected to the theoretically proposed TSMS at quadratic band‐touching points, evolve into broadened Hubbard‐like bands. These bands exhibit a dual character, comprising both localized and itinerant features. The itinerant bands interact with the topological bands, resulting in hole doping. The degree of this self‐doping is influenced by the strength of the temperature‐dependent TSMS, which leads to a temperature‐induced Lifshitz transition. The conventional understanding suggests that holes emerge due to intrinsic defects, and the Kondo hybridization involving R‐4f modifies low‐energy physics, thus affecting topological quasiparticles. These perspectives have been limited to modulating correlated topological materials. However, our study introduces an intrinsic mechanism of self‐doping based on the interaction between topological bands and correlated electrons, which influences exotic quantum phenomena in these materials. We theoretically propose two distinct mechanisms for Lifshitz transitions arising from the interplay between electronic correlations and topological band structures. These mechanisms may also be relevant to Lifshitz transitions observed in other topological materials, such as ${\mathrm{ZrTe}}_{2}$ [53] and ${\mathrm{WTe}}_{2}$ [64]. Moreover, our results may shed light on the origin of the Dirac node located below the Fermi level in Ce‐intercalated graphene [65]. This advancement should be instrumental in the design of topological superconductors and quantum computing devices within correlated topological systems.

## 8 Methods

### 8.1 LQSGW+DMFT Calculation

We used experimental lattice constants for the half‐Heusler compounds in space group $F\bar{4}3m$ (No.216): GdPtBi (a=6.680Å), YbPtBi (a=6.584Å), and YPtBi (a=6.640Å) [42]. Electronic structures were computed using the linearized quasiparticle self‐consistent GW method combined with dynamical mean‐field theory (LQSGW+DMFT) [41], a simplified realization of full GW+EDMFT [51, 58, 66, 67, 68]. The LQSGW approach provided the nonlocal self‐energy [69, 70], while the local component was corrected diagrammatically within DMFT [71, 72, 73]. The double‐counting energy and the dynamically screened Coulomb interaction tensor were computed explicitly (see Figure S1). Local self‐energies for the R‐4f and R‐5d shells were obtained by solving impurity models, with spin‐orbit coupling included in all calculations. Analytic continuation of the self‐energy from the imaginary‐ to the real‐frequency axis was performed using the maximum entropy method [74]; its validity in this context is demonstrated in Figure S2. Additional computational details are provided in the Supporting Information.

## Author Contributions

B.K. designed the project. B.K. performed the LQSGW+DMFT calculations and conducted the data analysis. All authors wrote the manuscript, discussed the results, and commented on the paper.

## Conflicts of Interest

The authors declare no conflicts of interest.

## Supporting information


**Supporting File**: advs74839‐sup‐0001‐SuppMat.pdf.

## Data Availability

The data that support the findings of this study are available from the corresponding authors upon reasonable request.
