# Supplementary material for: Lifshitz Transition in Correlated Topological Semimetals
Source: Adv Sci (Weinh). 2026 Mar 24;13(31):e21312. doi: 10.1002/advs.202521312 (PMC13588033; doi:10.1002/advs.202521312)
Supplement: Supplementary file 1 [file ADVS-13-e21312-s001.pdf]

Supplementary Information: Lifshitz transition in correlated topological semimetals

Byungkyun Kang[1,*], Myoung-Hwan Kim[2], Chul Hong Park[3], Anderson Janotti[4], and Eunja Kim[1]

[1] *Department of Physics 500 W University Ave, The University of Texas at El Paso, El Paso, Texas 79968, USA*

[2] *Department of Physics and Astronomy, Texas Tech University, Lubbock, Texas 79409, USA*

[3] *Quantum Matter Core-Facility and Research Center of Dielectric and Advanced Matter Physics, Pusan National University, Busan 46240, Republic of Korea*

[4] *Department of Materials Science and Engineering, University of Delaware, Newark, Delaware 19716, USA*

SUPPLEMENTARY METHODS

LQSGW+DMFT calculation

We adopted experimental lattice constants of space group $F\bar{4}3m$ (No. 216) (GdPtBi: a = 6.680 Å), (YbPtBi: a = 6.584 Å) and (YPtBi: a = 6.640 Å) [1]. We employed ab-$initio$ linearized quasiparticle self-consistent GW (LQSGW) method combined with dynamical mean field theory (DMFT) [2–4]. This LQSGW+DMFT is based on simplified full GW+DMFT approach [5–9]. The electronic structure was calculated using LQSGW approaches [10,11], and the local part of the GW self-energy was diagrammatically corrected within DMFT [12–14].We also explicitly calculated the double-counting energy and Coulomb interaction tensor. Local self-energies for R-4f and R-5d

in RPtBi were obtained by solving impurity models, with spin-orbital coupling included in all calculations.

LQSGW calculation

For LQSGW calculations, we use FlapwMBPT package [11], which is based on full-potential linearized augmented plane wave plus local orbital method. The Muffin-tin (MT) radius (R) are set (GdPtBi: 2.65 for Gd, 2.65 for Pt, 2.65 for Bi), (YbPtBi: 2.61 for Yb, 2.63 for Pt, 2.63 for Bi) and (YPtBi: 2.52 for Y, 2.65 for Pt, 2.65 for Bi) in Bohr radius. Wave functions are expanded in the MT spheres by spherical harmonics with l up to (GdPtBi: 6 for Gd, 6 for Pt, 6 for Bi), (YbPtBi: 6 for Yb, 6 for Pt, 6 for Bi) and (YPtBi: 6 for Y, 6 for Pt, 6 for Bi). In the interstitial region (IS), it is spanned by plane waves with the cutoff (K_{cut}) of $R_{\mathrm{U}}\times\mathrm{K}_{\mathrm{cut}}$= (GdPtBi: 7.97), (YbPtBi: 7.97) and (YPtBi: 7.62). Product basis set is spanned by spherical harmonics with l up to (GdPtBi: 6 for Gd, 6 for Pt, 6 for Bi), (YbPtBi: 6 for Yb, 6 for Pt, 6 for Bi) and (YPtBi: 6 for Y, 6 for Pt, 6 for Bi)in the MT spheres and by planewaves with the cutoff (G_{cut}) of $R_{\mathrm{U}}\times\mathrm{G}_{\mathrm{cut}}$= (GdPtBi: 12.8), (YbPtBi: 12.8) and (YPtBi: 12.2) in IS region. For both polarizability and self-energy calculations, we include all the unoccupied states. The Brillouin zone is sampled in $5 \times 5 \times 5$ grid.

Wannier function constructions

Wannier functions for (84 GdPtBi: Gd-s, Gd-p, Gd-d, Gd-f, Pt-s, Pt-p, Pt-d, Pt-f, Bi-s, Bi-p, Bi-d, Bi-f), (90 YbPtBi: Yb-s, Yb-p, Yb-d, Yb-f, Pt-s, Pt-p, Pt-d, Pt-f, Bi-s, Bi-p, Bi-d, Bi-f) and (54 YPtBi: Y-s, Y-p, Y-d, Pt-s, Pt-p, Pt-d, Bi-s, Bi-p, Bi-d) orbitals are constructed in a frozen energy

window between (GdPtBi: -12 eV to 10 eV), (YbPtBi: -12 eV to 10 eV) and (YPtBi: -15 eV to 8 eV) and with a disentanglement energy window of (GdPtBi: -12 eV to 50 eV), (YbPtBi: -12 eV to 50 eV) and (YPtBi: -15 eV to 50 eV). Initial trial orbitals are constructed by using Muffin-tin orbitals in LAPW basis set with well-defined angular momentum characters.

Constrained random phase approximation

The bosonic Weiss field $\widetilde{\mathcal{U}}$ for f orbitals and its Slater's integrals are computed within constrained random phase approximation (cRPA) [15,16]. Here we stress that the bosonic Weiss field $\widetilde{\mathcal{U}}$ from cRPA is a way to evaluate $\widetilde{\mathcal{U}}$ and not identical to $\widetilde{\mathcal{U}}$ from "ideal" fully self-consistent GW+EDMFT. Within cRPA, the bosonic Weiss field $\widetilde{\mathcal{U}}$ is obtained by separating out the RPA polarizability ($P_{\text{QP}}^{\text{low}}$) from the correlated states. In ComDMFT $P_{\text{QP}}^{\text{low}}$ is defined by identifying correlated bands [16–18]. These are bands having strong $4f$ orbital characters are chosen as correlated bands at each $\mathbf{k}$ point. The number of correlated bands is set to be the number of correlated orbitals for each spin.

Then, $P_{\text{QP}}^{\text{low}}$ is defined in the following way.

$$P_{\text{QP}}^{\text{low}}(\mathbf{r},\mathbf{r}',\mathbf{k},\mathrm{i}\nu_n) = -2\sum_{\mathbf{k}'}\sum_{N}^{\substack{\text{unoccupied}\\\text{correlated bands}}}\sum_{M}^{\substack{\text{occupied}\\\text{correlated bands}}} \psi_{N\mathbf{k}'}(\mathbf{r})\psi^*_{M\mathbf{k}'+\mathbf{k}}(\mathbf{r})\psi^*_{N\mathbf{k}'}(\mathbf{r}')\psi_{M\mathbf{k}'+\mathbf{k}}(\mathbf{r}')\frac{2(E_{N\mathbf{k}'}-E_{M\mathbf{k}'+\mathbf{k}})}{\nu_n^2-(E_{N\mathbf{k}'}-E_{M\mathbf{k}'+\mathbf{k}})^2}, \quad (1)$$

Here, $\psi_{N\mathbf{k}}(\mathbf{r})$ and $E_{n\mathbf{k}}$ are quasiparticle wave function and quasiparticle energy with a band index N and crystal momentum vector $\mathbf{k}$, respectively.

Using $P_{\mathrm{QP}}^{\mathrm{high}} = P_{\mathrm{QP}} - P_{\mathrm{QP}}^{\mathrm{low}}$ where P_{QP} is RPA polarizability, the partially-screened Coulomb interaction (W_r) is calculated by

$$W_r^{-1}(\mathbf{r},\mathbf{r}',\mathbf{k},\mathrm{i}\nu_n) = V^{-1}(\mathbf{r},\mathbf{r}',\mathbf{k}) - P_{\mathrm{QP}}^{\mathrm{high}}(\mathbf{r},\mathbf{r}',\mathbf{k},\mathrm{i}\nu_n). \quad (2)$$

Next, Slater's integrals (F^k) [19,20] are calculated by

$$\begin{aligned} F^k(\mathrm{i}\nu_n) =& \frac{1}{4C_k^l}\frac{4\pi}{2k+1} \sum_{m_1,m_2,m_3,m_4} \sum_{s,s'=\pm\frac{1}{2}} \sum_{m_1',m_2',m_3',m_4'} \sum_{i_1,i_2,i_3,i_4=\pm\frac{1}{2}} \\ & \langle Y_l^{m_1}|Y_k^{m_1-m_4}Y_l^{m_4}\rangle\langle Y_l^{m_2}Y_k^{m_2-m_3}|Y_l^{m_3}\rangle S_{m_1s,m_1'i_1}S_{m_2s',m_2'i_2}S^{-1}_{m_3s',m_3'i_3}S^{-1}_{m_4s,m_4'i_4} \\ & \int d\mathbf{r}d\mathbf{r}'W_r(\mathbf{r},\mathbf{r}',R=0,\mathrm{i}\nu_n)W^*_{R=0,m_1',i_1}(\mathbf{r})W^*_{R=0,m_2',i_2}(\mathbf{r}')W_{R=0,m_3'i_3}(\mathbf{r}')W_{R=0,m_4',i_4}(\mathbf{r}) \end{aligned} \quad (3)$$

where $W_{\mathbf{R},m,i}(\mathbf{r})$ is a Wannier function with angular part of spin-angular function, $S_{m_1s,m_1'i_1} = \langle l,m_1,\frac{1}{2},s|l+i_1,m_1'\rangle$, and $C_k^l = \frac{(2l+1)^4}{2k+1}\begin{bmatrix} l & k & l \\ 0 & 0 & 0 \end{bmatrix}, \begin{bmatrix} l & k & l \\ 0 & 0 & 0 \end{bmatrix}$ is the Racah-Wigner 3j-symbol.

Double counting energy

The electron self-energy included in both *ab initio* LQSGW and DMFT is the local Hartree term and the local GW term. They can be calculated as follows.

$$\widetilde{\Sigma}_{\mathrm{DC},i,j}(\mathrm{i}\omega_n) = 2\sum_{k,l=m_l'}^{\text{correlated-orbitals}} \widetilde{G}_{l,k}(\tau=0^-)\widetilde{\mathcal{U}}_{iklj}(\mathrm{i}\nu=0) - \sum_{k,l}^{\text{correlated-orbitals}} \int d\tau \widetilde{G}_{l,k}(\tau)\widetilde{W}_{ikjl}(\tau)e^{\mathrm{i}\omega_n\tau}. \quad (4)$$

where i,j,k and l are orbital indices. $\widetilde{G}$ is the local Green's function. $\widetilde{\mathcal{U}}$ is constructed by using Slater's integrals in Supplementary Equation (3).

$$\widetilde{\mathcal{U}}_{i,j,k,l}(\mathrm{i}\nu_n) = \sum_{m'_1 m'_2, m'_3 m'_4} S_{i,m_1} S_{j,m_2} S^{-1}_{k,m_3} S^{-1}_{l,m_4} \sum_{k=0}^{2l,even} \frac{4\pi}{2k+1} \langle Y_l^{m'_1} | Y_k^q Y_l^{m'_4} \rangle \langle Y_l^{m'_2} Y_k^q | Y_l^{m'_3} \rangle F^k(\mathrm{i}\nu_n). \tag{5}$$

Here, we assume that the frequency-dependent interaction is of the form

$$\widetilde{\mathcal{U}}_{ijkl}(\mathrm{i}\nu_n) = \widetilde{U}_{ijkl} + F^0(\mathrm{i}\nu_n)\delta_{il}\delta_{jk}, \tag{6}$$

that is, only the dynamical screening of the Slater-Condon parameter F^0 is taken into account. The other Slater-Condon parameters, which define $\widetilde{U}_{ijkl}$, are frequency independent and approximated by their value at $\nu_n = \infty$. $\widetilde{W}$ is the local screened Coulomb interaction given by

$$\widetilde{W}_{ikjl}(\mathrm{i}\nu_n) = \widetilde{\mathcal{U}}_{ikjl}(\mathrm{i}\nu_n) + \sum_{mnpq}^{\text{correlated-orbitals}} \widetilde{\mathcal{U}}_{imnl}(\mathrm{i}\nu_n) \widetilde{P}_{mpqn}(\mathrm{i}\nu_n) \widetilde{W}_{pkjq}(\mathrm{i}\nu_n), \tag{7}$$

where $\widetilde{P}$ is the local polarizability and it is calculated as

$$\widetilde{P}_{mpqn}(\mathrm{i}\nu_n) = \int d\tau \widetilde{G}_{n,p}(\tau) \widetilde{G}_{q,m}(-\tau) e^{\mathrm{i}\nu_n \tau}. \tag{8}$$

DMFT self-consistent equation

At each iteration of the fermionic DMFT self-consistent loop, the fermionic Weiss-field is constructed in the following way.

$$\widetilde{\mathcal{G}} = \left(\left(\frac{1}{N_{\mathbf{k}}} \sum_{\mathbf{k}} f^{\dagger}_{\mathbf{k}} G(\mathbf{k}, \mathrm{i}\omega_n) f_{\mathbf{k}} \right)^{-1} + \widetilde{\Sigma}_{\mathrm{imp}} \right)^{-1} \tag{9}$$

Here $f_{\mathbf{k}}$ is the fermionic projection operator to correlation orbitals (R-4f and R-5d orbitals) and given by $f_{\mathbf{k}} = \langle \mathbf{r}|W_{i\mathbf{k}}\rangle$ where $|W_{i\mathbf{k}}\rangle = \frac{1}{\sqrt{N_{\mathbf{k}}}} \sum_{\mathbf{R}} |W_{i\mathbf{R}}\rangle e^{i\mathbf{k}\cdot\mathbf{R}}$. $\widetilde{\Sigma}_{\mathrm{imp}}$ is impurity self-energy from impurity solver. Within *ab initio* LQSGW+DMFT, lattice Green's function is calculated by embedding impurity self-energy into the LQSGW Green's function

$$G^{-1}(\mathbf{k}, \mathrm{i}\omega_n) = \mathrm{i}\omega_n - H^{nl}_{\mathrm{QP}}(\mathbf{k}) - f_{\mathbf{k}} \widetilde{\Sigma}_{\mathrm{imp}}(\mathrm{i}\omega_n) f^{\dagger}_{\mathbf{k}}, \tag{10}$$

where H^{nl}_{QP} is non-local LQSGW Hamiltonian[2], in which double-counting self-energy is compensated up to linear order in frequency.

$$H^{nl}_{\mathrm{QP}}(\mathbf{k}) = \sqrt{Z^{-1}_{\mathrm{DC}}(\mathbf{k})} H_{\mathrm{QP}} \sqrt{Z^{-1}_{\mathrm{DC}}(\mathbf{k})} - f_{\mathbf{k}} \widetilde{\Sigma}_{\mathrm{DC}}(\omega = 0) f^{\dagger}_{\mathbf{k}}. \tag{11}$$

Here, H_{QP} is Wannier interpolated LQSGW Hamiltonian into $6 \times 6 \times 6$ k-grid. $Z^{-1}_{\mathrm{DC}}(\mathbf{k}) = 1 - f_{\mathbf{k}} \left(\partial \widetilde{\Sigma}_{\mathrm{DC}}(\omega = 0) / \partial \mathrm{i}\omega_n \right) f^{\dagger}_{\mathbf{k}}$.

ComDMFT necessitates the solution of an impurity model action. In ComDMFT, hybridization-expansion continuous-time quantum Monte Carlo (CTQMC) is adopted. CTQMC is a stochastic approach to obtain numerically exact solutions of an impurity model. An impurity model consists of a small interacting system, the impurity, immersed in a bath of non-interacting electrons. The action of the impurity model relevant for GW+DMFT reads

$$
\begin{aligned}
S = &-\iint_0^\beta \sum_{ij} c_i^\dagger(\tau)\widetilde{\mathcal{G}}_{ij}^{-1}(\tau-\tau')c_j(\tau')d\tau d\tau' \\
&+\frac{1}{2}\iint_0^\beta \sum_{ijkl} c_i^\dagger(\tau)c_j^\dagger(\tau')\widetilde{\mathcal{U}}_{ijkl}(\tau-\tau')c_k(\tau')c_l(\tau)d\tau d\tau',
\end{aligned}
\tag{12}
$$

where $c_i^\dagger$ creates an electron in the generalized orbital i (which includes both spin and orbital degrees of freedom), β is the inverse temperature, $\widetilde{\mathcal{G}}_{ij}$ is the fermionic Weiss field in Supplementary Equation (9) and $\widetilde{\mathcal{U}}_{ijkl}$ in Supplementary Equation (5).

We assume that the frequency-dependent interaction is of the form

$$
\widetilde{\mathcal{U}}_{ijkl}(\mathrm{i}\nu_n) = \widetilde{U}_{ijkl} + F^0(\mathrm{i}\nu_n)\delta_{il}\delta_{jk}, \tag{13}
$$

that is, only the dynamical screening of the Slater-Condon parameter F^0 is taken into account, for the simplicity in the numerical algorithm based on hybridization-expansion CTQMC. The other Slater-Condon parameters, which define $\widetilde{U}_{ijkl}$, are frequency independent and approximated by their value at $\nu_n = \infty$.

SUPPLEMENTARY FIGURES

On-site Coulomb interaction

The constrained random phase approximation (cRPA) [15] was employed to determine the frequency-dependent on-site Coulomb interaction, U_{C}, as well as the exchange interaction, J_{H}.

Utilizing quasiparticle wave functions and quasiparticle energies obtained from the linearized quasiparticle GW approximation, we computed the partially screened on-site Coulomb interaction

for Slater's integrals, as detailed in the "Constrained Random Phase Approximation" section of the Supplemental Information. This methodology does not require predefined assumptions regarding the R-4f and Y-4d electronic configurations, thereby enabling fully ab initio calculations. As a result, the static U_{C} values for Gd-4f, Yb-4f, and Y-4d were determined to be 3.4, 3.1, and 1.9 eV in GdPtBi, YbPtBi, and YPtBi, respectively.

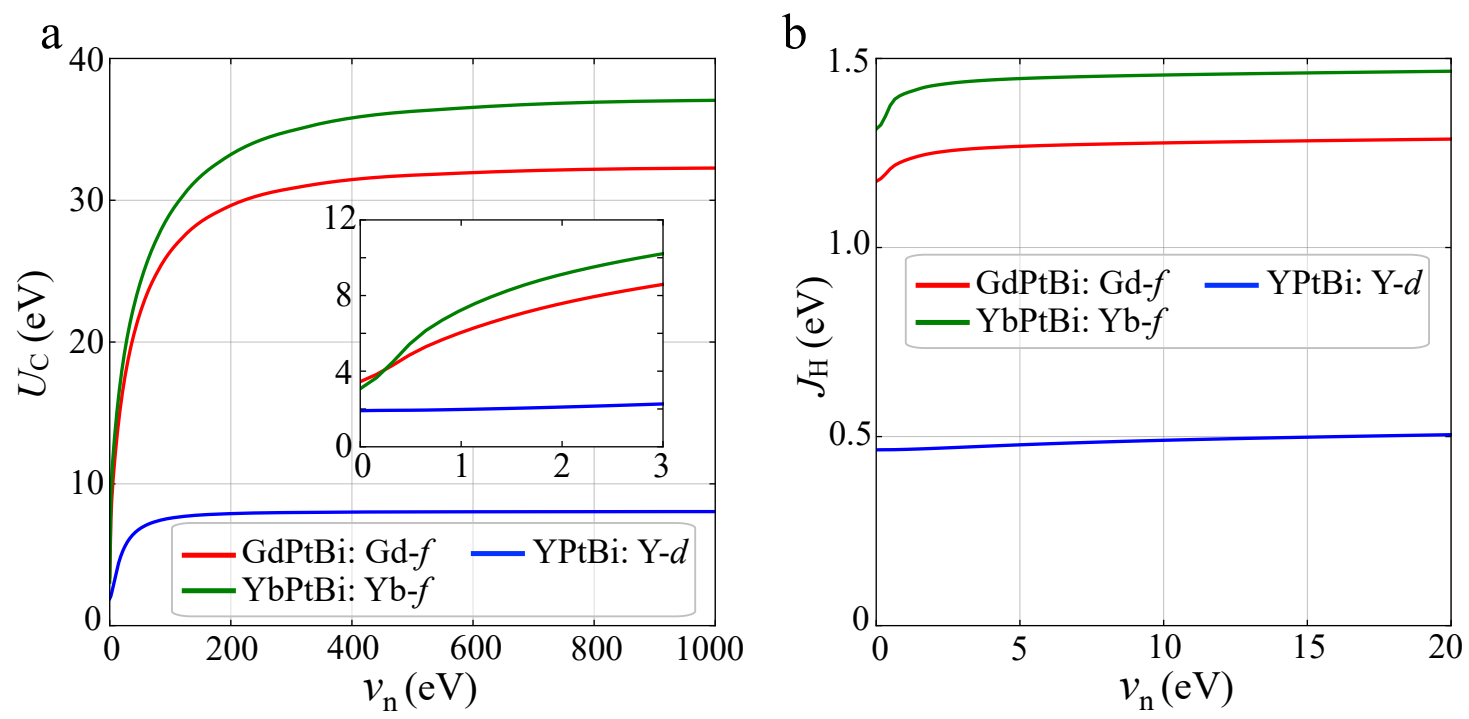

Supplementary Figure 1: **calculated on-site Coulomb interaction. a**, Coulomb interaction U_{C} **b**, Exchange interaction J_{H} for Gd-4f in GdPtBi, Yb-4f in YbPtBi, and Y-4d in YPtBi. In (**a**), the inset shows magnified views of U_{C} in the low frequency range.

Justification of analytic continuation

We show that the divergent self-energy observed on the imaginary frequency axis naturally arises as a solution of the LQSGW+DMFT formalism. Importantly, this divergence is intrinsic to the solution itself and does not result from any post-processing or analytic continuation procedure. Nevertheless, analytic continuation appropriately maps this divergence to the real frequency axis, enabling us to capture the corresponding features of the self-energy in the real frequency domain.

We performed analytical continuation using the maximum entropy method [M. Jarrell, J.E.

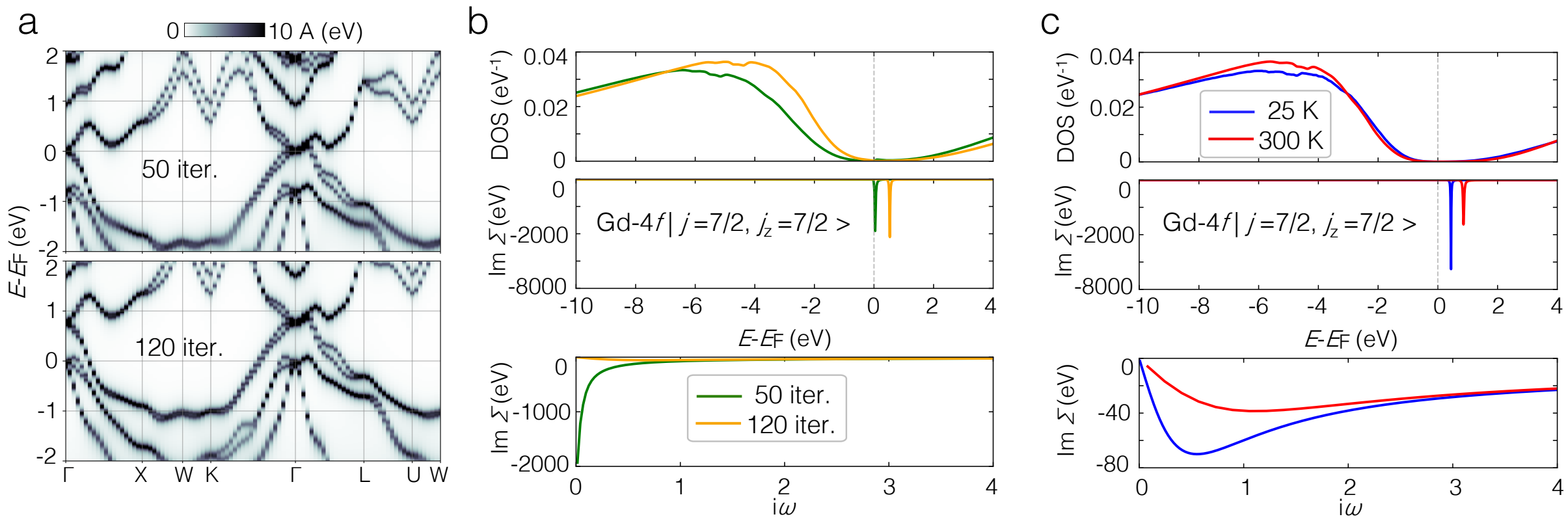

Supplementary Figure 2: **Electronic structure of GdPtBi.** **a**, Spectral functions after 50 iterations (upper panel) and 120 iterations (lower panel) of DMFT self-consistent loops at 300 K. **b**, Projected density of states (top panel), and the imaginary part of the self-energy of the Gd-4f state of the $|j = 7/2, j_z = 7/2\rangle$ on the real (middle panel) and imaginary (bottom panel) frequency axes at 300 K. **c**, Projected density of states (top panel) and the imaginary part of the self-energy for the Gd-4f state of the $|j = 7/2, j_z = 7/2\rangle$ on the real (middle panel) and imaginary (bottom panel) frequency axes at 25 K and 300 K.

Gubernatis, Phys. Rep. 269 (3) (1996) 133–195] [21].

To validate the analytical continuation procedure, we examine the self-energy on both imaginary and real frequency axes. Figures 2a and b present the spectral functions, density of states, and self-energy calculated at real and imaginary frequencies after 50 and 120 iterations of the DMFT self-consistency cycle for GdPtBi. While the DMFT loop reaches convergence at 120 iterations, we also analyze the self-energy after 50 iterations—corresponding to an intermediate stage of the self-consistency process—for comparison with the fully converged results.

After 50 iterations, a singularity appears at the Fermi level, resulting in a TSMS (Topological Semimetallic State) at the Fermi level on the real frequency axis. On the imaginary frequency axis, this TSMS manifests as a pole at ($i\omega = 0$), analogous to the divergent self-energy observed in a Mott insulator, where the pole also occurs at the Fermi level ($i\omega = 0$). Our analytic continuation is able to capture this strong divergence of approximately (-2000) eV at $i\omega = 0$ on the imaginary frequency axis as a divergent peak of similar magnitude (approximately (-2000) eV) at the Fermi level on the real frequency axis.

At 120 iterations, a topological singularity emerges slightly above the Fermi level. This results in a divergent TSMS above the Fermi level on the real frequency axis, and manifests as a pole in the self-energy above $i\omega = 0$ on the imaginary frequency axis. Compared to the self-energy observed at 50 iterations, our analytic continuation successfully captures the energy shift of this pole away from the Fermi level, while showing a similar magnitude for the self-energy on the real frequency axis.

These results therefore suggest that our analytic continuation successfully captures the relevant physics when mapping from the imaginary to the real frequency axis. This can also be seen in Fig. 2c. On the imaginary frequency axis, the pole of the TSMS approaches $i\omega = 0$ and its magnitude increases upon cooling. On the real frequency axis, the TSMS correspondingly moves closer to the Fermi level, with an enhanced magnitude.

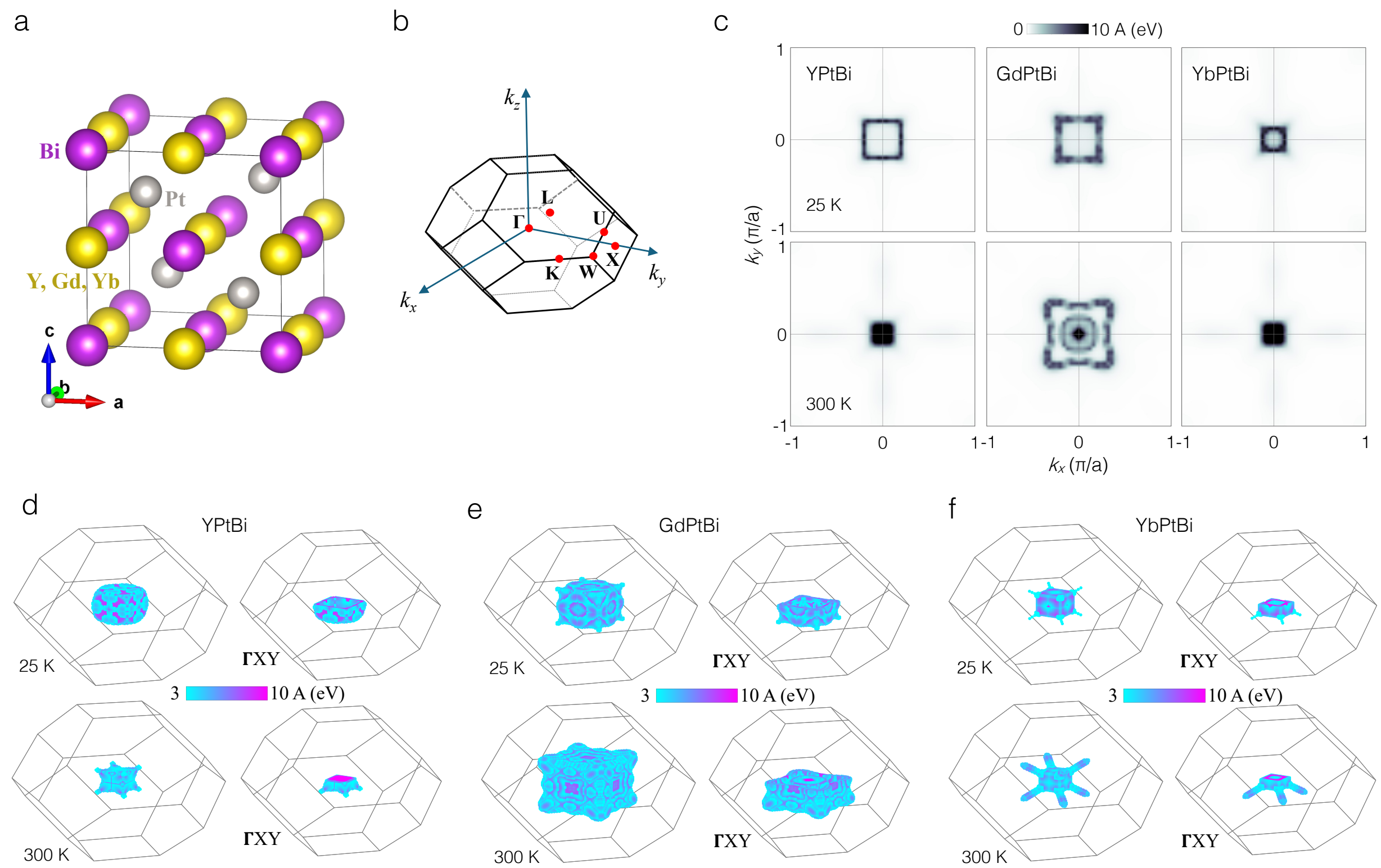

Supplementary Figure 3: **Fermi surfaces of RPtBi (R=Y,Gd,Yb).** (a) Crystal structure. (b) The first Brillouin zone. (c) Calculated Fermi surfaces with k_z = 0 for 300 K and 25 K. The Fermi surface (left) and upper of ΓXY plane removed Fermi surface (right) in the Brillouin Zone for (d) YPtBi, (e) GdPtBi, and (f) YbPtBi at 300 K and 25 K.

Lifshitz transition of the Fermi surface

Figure 3a, b, and c show the crystal structure, the first Brillouin zone, and the calculated Fermi surfaces of RPtBi (R = Gd, Yb, and Y) at $k_z = 0$ for temperatures of 300 K and 25 K, respectively. For YPtBi, the Fermi surface at 300 K is a small, point-like feature, which evolves into an unfilled square shape at 25 K due to the emergence of hole bands upon cooling. In contrast, GdPtBi exhibits a large square-like Fermi surface at 300 K, which shrinks at lower temperatures as the quadratic

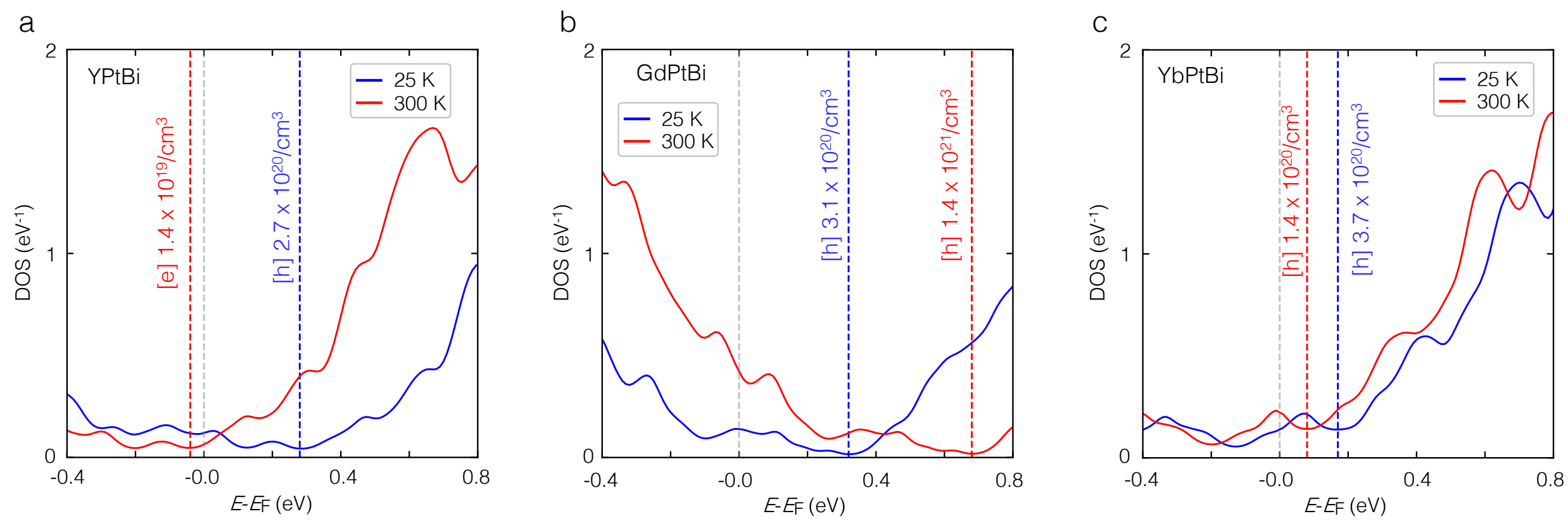

Supplementary Figure 4: **Carrier density.** Calculated carrier densities obtained by integrating the density of states from the chemical potential to the quadratic band-touching points at 25 K and 300 K for **a** YPtBi, **b** GdPtBi, and **c** YbPtBi. The chemical potential is set to zero, as indicated by the gray dashed line. Quadratic band-touching points are indicated by blue (25 K) and red (300 K) dashed lines.

band-touching point of the topological hole bands approaches the chemical potential. Meanwhile, YbPtBi shows only minor Fermi surface reconstruction with decreasing temperature, a behavior attributed to enhanced Yb-$4d$ electron correlations.

Figure 3d, e, and f display the calculated three-dimensional Fermi surfaces of RPtBi (R = Y, Gd, and Yb) at temperatures of 300 K and 25 K. To visualize the Fermi surfaces, we plot the spectral function $A(\mathbf{k}, \omega)$ at finite temperature, where $\mathbf{k}$ values are sampled over a grid covering the Brillouin Zone and ω is fixed at the Fermi level. All three compounds exhibit three-dimensional Fermi surface characteristics, with notable changes in shape between 300 K and 25 K, indicative of a Lifshitz transition. Specifically, for YPtBi at 300 K, the Fermi surface consists of small,

closely packed cube-like features and eight pairs of small, teardrop-shaped regions of spectral weight. At 25 K, these features evolve into larger cube-shaped hole pockets. In GdPtBi, the Fermi surface volume is the largest among the three compounds, as the topological singular point lies far from the Fermi level. At 300 K, two large cube-shaped Fermi surfaces are observed, which evolve into a single, smaller cube-shaped hole pocket at 25 K. In the case of YbPtBi, since the Lifshitz transition is less pronounced, a subtle change preserves the quadratic band-touching point near the Fermi level at both temperatures. The resulting Fermi surfaces show small cube-shaped hole pockets and eight pairs of small, teardrop-shaped electron pockets at the corners of the hole pockets, in agreement with previously reported density functional theory calculations [22].

To provide further evidence for the Lifshitz transition, we calculated the carrier density based on the density of states (DOS) and electronic band structure, as shown in Fig. **4**. Our method differs from that of Pavlosiuk *et al.*[23], who estimated the carrier concentration in GdPtBi using $n = V_F/(4\pi^3)$, where V_F is the volume of the Fermi pocket, with the assumption of a spherical pocket shape. In contrast, our approach involves direct integration of the DOS from the chemical potential to the band-touching point, a technique previously shown to yield accurate carrier densities for the topological semimetal LaAlGe [24].

For YPtBi and GdPtBi, we observe significant changes in carrier density associated with the Lifshitz transition, along with intrinsically low carrier densities. For YPtBi, at 300K, the quadratic band-touching point lies just below the Fermi level, indicating electron-like charge carriers. However, upon cooling, the Lifshitz transition leads to a dominance of holes, with a calculated hole

density of $2.7 \times 10^{20}/\mathrm{cm}^{-3}$ at 25K—consistent with experimentally measured values for YPtBi, which range from 10^{18} to $10^{21}/\mathrm{cm}^{-3}$ [25,26].

In the case of GdPtBi, we obtain a hole carrier density of $1.4 \times 10^{21}/\mathrm{cm}^{-3}$ at 300K, the largest among the three compounds, as the chemical potential is well separated from the quadratic band-touching point. This decreases to $3.1 \times 10^{20}/\mathrm{cm}^{-3}$ following the Lifshitz transition. The calculated hole density is somewhat higher than the Hall-measured densities, which are on the order of $10^{20}/\mathrm{cm}^{-3}$ at 300 K and decrease to $\sim 10^{18}/\mathrm{cm}^{-3}$ at 50 K[27].

For YbPtBi, despite the likely presence of two types of carriers affecting Hall measurements [28,29], our calculated hole densities—$1.4 \times 10^{20}/\mathrm{cm}^{-3}$ at 300 K and $3.7 \times 10^{20}/\mathrm{cm}^{-3}$ at 25 K—are consistent with the dominant experimentally measured hole carrier density of $6 \times 10^{20}/\mathrm{cm}^{-3}$ [30].

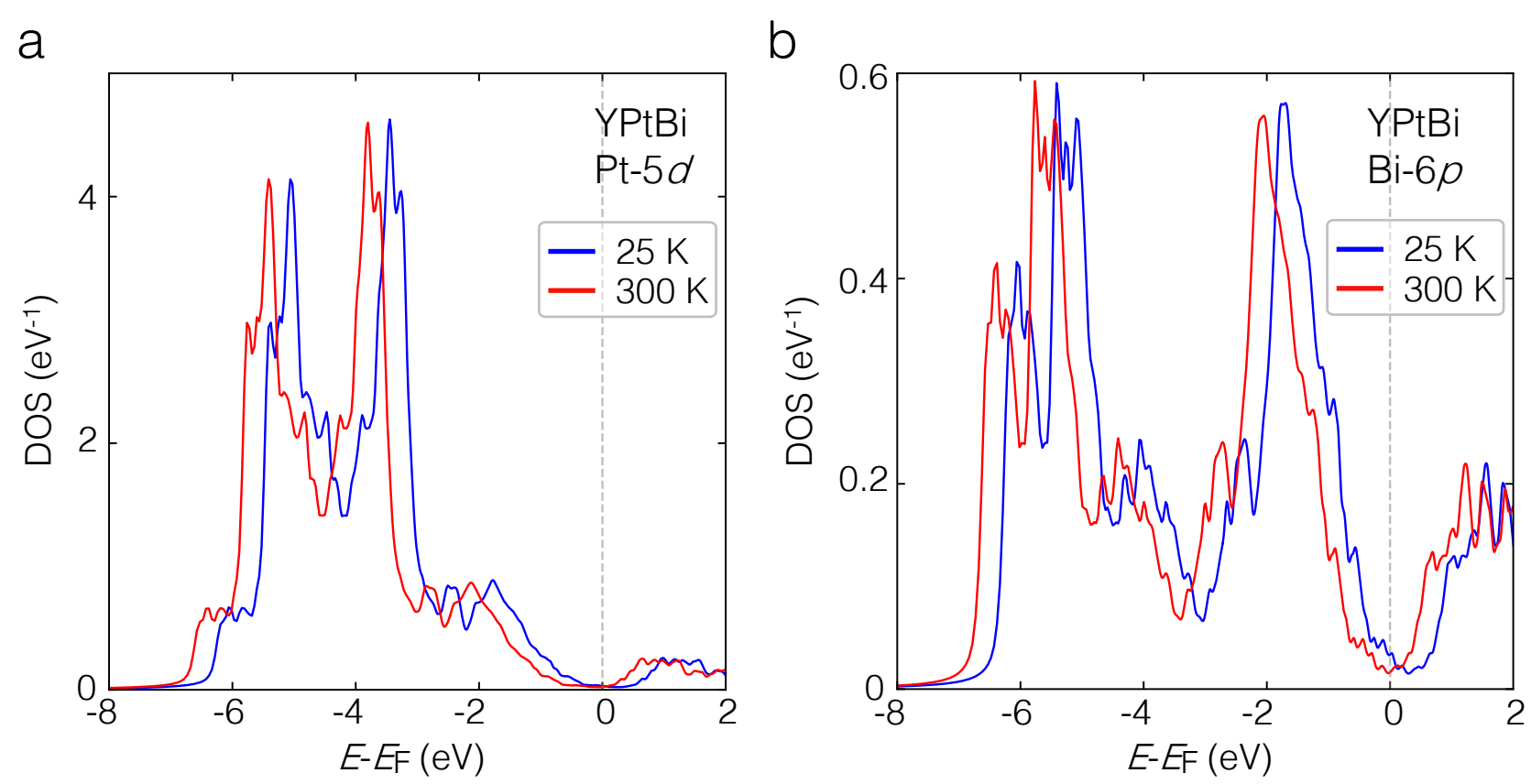

Supplementary Figure 5: **Orbital-projected density of states (DOS) of YPtBi. a**, Projected DOS of Pt-5d at 25 K and 300K. **b**, Projected DOS of Bi-6p at 25 K and 300K.

Charge transfer in YPtBi

The LQSGW+DMFT methodology employs a DMFT self-consistency loop, wherein the Weiss field ($\widetilde{\mathcal{G}}$) is generated via the `ComLowH` module, and both the chemical potential (μ) and impurity self-energy are subsequently determined using `ComCTQMC` based on the computed $\widetilde{\mathcal{G}}$ [4]. Within `ComLowH`, the lattice Green's function (G) is calculated by explicitly incorporating the non-local LQSGW Hamiltonian (H^{nl}_{QP}) and local impurity self-energy ($\widetilde{\Sigma}_{imp}$), which also accounts for the chemical potential, and is subsequently used to obtain the Weiss field $\widetilde{\mathcal{G}}$. The iterative combination of `ComLowH` and `ComCTQMC` facilitates a self-consistent determination of both the chemical potential and self-energy by treating the local DMFT Hamiltonian and the non-local LQSGW Hamiltonian within a unified lattice Green's function formalism. This approach enables charge transfer between eigenstates, while ensuring that the total number of occupied electrons remains conserved through convergence of the chemical potential within the unified lattice Green's function

and occupancy changes reflecting a physical mechanism based on non-local and local combined Hamiltonian.

The occupation values for correlated orbitals, such as R-d and R-f, are obtained by applying the occupation operator in Matsubara frequency within the CTQMC solver as part of the LQSGW+DMFT framework. However, the measurement of occupations for uncorrelated orbitals, such as Bi-p, within the full space of the lattice Green's function has not yet been implemented in LQSGW+DMFT.

Occupancy values estimated from the density of states (DOS) via analytic continuation are subject to errors. Moreover, the use of a coarse energy grid in real frequency axis calculations, especially far from the Fermi level, may introduce additional inaccuracies in the estimation of the occupancy from the DOS.

For these reasons, we are unable to provide reliable occupancy values for uncorrelated orbitals. Instead, we analyze the shape of the orbital-projected DOS to identify charge transfer from Y-d to Pt-d and Bi-p. In YPtBi, the valence bands in the range from -8 eV to the Fermi level are primarily composed of Pt-5d and Bi-6p states, with a smaller contribution from Y-4d. As illustrated in Fig. **5**, a comparison of the DOS at 300 K and 25 K reveals that the peak height of the Pt-5d DOS around -4 eV and -2 eV increases slightly at 25 K. Furthermore, the DOS of Bi-6p in the range from -3 eV to the Fermi level also increases at 25 K. In contrast, the Y-4d occupancy decreases at 25 K. These observations indicate a charge transfer from Y-4d orbitals to Pt-5d and Bi-6p orbitals upon cooling.

SUPPLEMENTARY REFERENCES

1. Haase, M. G., Schmidt, T., Richter, C. G., Block, H. & Jeitschko, W. Equiatomic rare earth (Ln) transition metal antimonides LnTSb (T= Rh, lr) and bismuthides LnTBi (T= Rh, Ni, Pd, Pt). *J. Solid State Chem.* **168**, 18–27 (2002). URL https://doi.org/10.1006/jssc.2002.9670.

2. Tomczak, J. M. QSGW+ DMFT: an electronic structure scheme for the iron pnictides and beyond. *J. Phys. Conf. Ser.* **592**, 012055 (2015).

3. Choi, S., Kutepov, A., Haule, K., van Schilfgaarde, M. & Kotliar, G. First-principles treatment of Mott insulators: linearized QSGW+DMFT approach. *npj Quantum Mater.* **1**, 16001 (2016). URL https://doi.org/10.1038/npjquantmats.2016.1.

4. Choi, S., Semon, P., Kang, B., Kutepov, A. & Kotliar, G. ComDMFT: A massively parallel computer package for the electronic structure of correlated-electron systems. *Comput. Phys. Commun.* **244**, 277–294 (2019). URL https://www.sciencedirect.com/science/article/pii/S0010465519302140.

5. Sun, P. & Kotliar, G. Extended dynamical mean-field theory and GW method. *Phys. Rev. B* **66**, 085120 (2002). URL https://doi.org/10.1103/PhysRevB.66.085120.

6. Biermann, S., Aryasetiawan, F. & Georges, A. First-Principles Approach to the Electronic Structure of Strongly Correlated Systems: Combining the G W Approximation and Dynamical Mean-Field Theory. *Phys. Rev. Lett.* **90**, 086402 (2003). URL https://doi.org/10.1103/PhysRevLett.90.086402.

7. Nilsson, F., Boehnke, L., Werner, P. & Aryasetiawan, F. Multitier self-consistent $GW +$ EDMFT. *Phys. Rev. Mater.* **1**, 043803 (2017). URL https://link.aps.org/doi/10.1103/PhysRevMaterials.1.043803.

8. Kang, B. *et al.* ComDMFT v.2.0: Fully self-consistent ab initio gw+edmft for the electronic structure of correlated quantum materials. *Comput. Phys. Commun.* **308**, 109447 (2025). URL https://www.sciencedirect.com/science/article/abs/pii/S0010465524003709.

9. Kang, B., Kim, M., Park, C. H. & Janotti, A. Mott-Insulator State of FeSe as a Van der Waals 2D Material Is Unveiled. *Phys. Rev. Lett.* **132**, 266506 (2024). URL https://link.aps.org/doi/10.1103/PhysRevLett.132.266506.

10. Kutepov, A., Haule, K., Savrasov, S. Y. & Kotliar, G. Electronic structure of Pu and Am metals by self-consistent relativistic GW method. *Phys. Rev. B* **85**, 155129 (2012). URL https://link.aps.org/doi/10.1103/PhysRevB.85.155129.

11. Kutepov, A., Oudovenko, V. & Kotliar, G. Linearized self-consistent quasiparticle GW method: application to semiconductors and simple metals. *Comput. Phys. Commun.* **219**, 407–414 (2017). URL https://www.sciencedirect.com/science/article/pii/S0010465517301947.

12. Georges, A., Kotliar, G., Krauth, W. & Rozenberg, M. J. Dynamical mean-field theory of strongly correlated fermion systems and the limit of infinite dimensions. *Rev. Mod. Phys.* **68**, 13–125 (1996). URL https://link.aps.org/doi/10.1103/RevModPhys.68.13.

13. Metzner, W. & Vollhardt, D. Correlated lattice fermions in $d = \infty$ dimensions. *Phys. Rev. Lett.* **62**, 324–327 (1989). URL https://link.aps.org/doi/10.1103/PhysRevLett.62.324.

14. Georges, A. & Kotliar, G. Hubbard model in infinite dimensions. *Phys. Rev. B* **45**, 6479–6483 (1992). URL https://link.aps.org/doi/10.1103/PhysRevB.45.6479.

15. Aryasetiawan, F. *et al.* Frequency-dependent local interactions and low-energy effective models from electronic structure calculations. *Phys. Rev. B* **70**, 195104 (2004). URL https://link.aps.org/doi/10.1103/PhysRevB.70.195104.

16. Aryasetiawan, F., Karlsson, K., Jepsen, O. & Schönberger, U. Calculations of Hubbard U from first-principles. *Phys. Rev. B* **74**, 125106 (2006). URL https://link.aps.org/doi/10.1103/PhysRevB.74.125106.

17. Miyake, T. & Aryasetiawan, F. Screened Coulomb interaction in the maximally localized Wannier basis. *Phys. Rev. B* **77**, 085122 (2008). URL https://link.aps.org/doi/10.1103/PhysRevB.77.085122.

18. Werner, P. *et al.* Satellites and large doping and temperature dependence of electronic properties in hole-doped $BaFe_2As_2$. *Nat. Phys.* **8**, 331–337 (2012). URL http://www.nature.com/nphys/journal/v8/n4/abs/nphys2250.html.

19. Slater, J. C. *Quantum Theory of Atomic Structure* (McGraw-Hill, 1960). Google-Books-ID: W8NEAAAAIAAJ.

20. Sugano, S. *Multiplets of Transition-Metal Ions in Crystals* (Elsevier, 2012). URL http://www.sciencedirect.com/science/bookseries/00798193/33.

21. Jarrell, M. & Gubernatis, J. E. Bayesian inference and the analytic continuation of imaginary-time quantum Monte Carlo data. *Phys. Rep.* **269**, 133–195 (1996). URL https://doi.org/10.1016/0370-1573(95)00074-7.

22. Mun, E. *et al.* Quantum oscillations in the heavy-fermion compound YbPtBi. *Phys. Rev. B* **92**, 085135 (2015). URL https://doi.org/10.1103/PhysRevB.92.085135.

23. Pavlosiuk, O. *et al.* Tuning of anomalous magnetotransport properties in half-Heusler topological semimetal GdPtBi. *Mater. Horiz.* (2025). URL https://doi.org/10.1039/D4MH01875C.

24. Kim, I., Kang, B., Kim, H. & Choi, M. Crystallographic defects in Weyl semimetal LaAlGe. *Phys. Rev. Mater.* **8**, 054203 (2024). URL https://doi.org/10.1103/PhysRevMaterials.8.054203.

25. Butch, N. P., Syers, P., Kirshenbaum, K., Hope, A. P. & Paglione, J. Superconductivity in the topological semimetal YPtBi. *Phys. Rev. B* **84**, 220504 (2011). URL https://doi.org/10.1103/PhysRevB.84.220504.

26. Shirokura, T. & Hai, P. N. Giant spin Hall effect in half-Heusler alloy topological semimetal YPtBi grown at low temperature. *AIP Adv.* **12** (2022). URL https://doi.org/10.1063/5.0117613.

27. Hirschberger, M. *et al.* The chiral anomaly and thermopower of Weyl fermions in the half-Heusler GdPtBi. *Nat. Mater.* **15**, 1161–1165 (2016). URL http://dx.doi.org/10.1038/NMAT4684.

28. Mun, E. *et al.* Magnetic-field-tuned quantum criticality of the heavy-fermion system YbPtBi. *Phys. Rev. B* **87**, 075120 (2013). URL https://doi.org/10.1103/PhysRevB.87.075120.

29. Schilling, M. *et al.* Two-channel conduction in ybptbi. *Phys. Rev. B* **95**, 155201 (2017). URL https://doi.org/10.1103/PhysRevB.95.155201.

30. Hundley, M. F., Thompson, J. D., Canfield, P. & Fisk, Z. Electronic transport properties of the semimetallic heavy fermion YbBiPt. *Phys. Rev. B* **56**, 8098 (1997). URL https://doi.org/10.1103/PhysRevB.56.8098.
